# Barriers and Facilitators to Housing With Services: Lessons From The Right Care, Right Place, Right Time Program

**DOI:** 10.1093/geroni/igaa057.103

**Published:** 2020-12-16

**Authors:** Edward Miller, Elizabeth Simpson, Pamela Nadash, Natalie Shellito, Taylor Jansen, Yan Lin, Marc Cohen

**Affiliations:** University of Massachusetts Boston, Boston, Massachusetts, United States

## Abstract

The Right Care, Right Place, Right Time initiative (R3) was developed to enable seniors to remain at home as long as possible, while reducing health care costs. It was implemented in four senior housing communities in the Greater Boston area, and consists of two on-site wellness teams (wellness nurse, wellness coordinator), each responsible for about 200 participants across two housing sites. This study aimed to understand barriers and facilitators to implementing R3. Data derived from 31 semi-structured interviews with R3 staff, housing personnel, and community partners (e.g., first responders), as well as 150 key program documents. Facilitating factors in implementing R3 included: top-level management support; formal and informal mechanisms of communication between wellness team members and building staff; substantial discretion, flexibility, and creativity provided to wellness team members; and daily ambulance reports from first responders. Barriers to implementing R3 included: impediments to resident recruitment/engagement; initial role confusion between wellness team members and existing building staff; limited wellness team time at individual intervention sites; challenges establishing systematic relationships with case management staff from the hospitals, AAAs, and insurance companies; and the decentralized approach to data tracking and information exchange. This study suggests several lessons for implementing housing with services initiatives such as R3. Top-level support and buy-in at the organizational level is essential to program development and implementation. Despite early challenges, key program elements can improve over time (communication, data processes, role clarity). Establishing trust with both R3 participants and housing staff is key to building relationships that promote program success.

